# Disparities in Secure Messaging Uptake Between Patients and Physicians: Longitudinal Analysis of Two National Cross-Sectional Surveys

**DOI:** 10.2196/12611

**Published:** 2020-05-01

**Authors:** Dawn M Heisey-Grove, Henry J Carretta

**Affiliations:** 1 Department of Clinical Quality and Informatics Health Technical Center MITRE Corporation McLean, VA United States; 2 College of Medicine Florida State University Tallahassee, FL United States

**Keywords:** electronic mail, health communication, electronic health records

## Abstract

**Background:**

Emails securely exchanged between patients and clinicians offer the promise of improved access to care and indirectly improved health outcomes. Yet research to date is mixed on who—among both patients and clinicians—is using secure messaging.

**Objective:**

Using data from two large nationally representative cross-sectional surveys, this study aimed to compare the prevalence of secure messaging use among patients and their access to the functionality through their physicians, and to explore the clinical practice and physician characteristics and patient sociodemographic characteristics associated with the use of secure messaging.

**Methods:**

We conducted regression analyses to identity statistical associations between self-reported secure messaging use and access, and the patient, practice, and physician characteristics from the National Health Interview Survey (NHIS) and the National Ambulatory Medical Care Survey (NAMCS). The NHIS data collected between 2013 and 2018, with approximately 150,000 adult individuals, were used to evaluate patient characteristics associated with email communication with clinicians. The NAMCS data included 7340 physicians who reported on secure messaging use between 2013 and 2016 and provided context on physician specialty, use of certified health information technology (IT), and practice size and ownership associated with secure messaging access and use.

**Results:**

By 2016, two-thirds of ambulatory care visits were conducted by a physician who reported using secure messaging, up from 40.70% in 2013. The percentage of US residents who reported sending an email to their clinician, however, only increased from 7.22% to 16.67% between 2013 and 2018. We observed a strong positive association between certified health IT use and secure messaging use (odds ratio [OR] 11.46, 95% CI 7.55-17.39). Individuals who were black, had lower levels of education, had Medicaid or other public payer insurance, or those who were uninsured had reduced odds for using email to communicate with clinicians. No differences were observed in secure messaging use based on physician specialty, but significant differences were observed by practice size (OR 0.46, 95% CI 0.35-0.60 in solo practices vs nonsolo practices) and practice ownership (*P*<.001 for the different categories).

**Conclusions:**

This study is the first to use two large nationally representative surveys to produce longitudinal estimates on the access and use of patient-clinician email communication in the United States. The survey findings complement each other: one provides the patient perspective of their use and the other indicates potential patient access to secure messaging based on the use of the functionality by the physicians providing treatment. This study provides nationally representative data on the characteristics of patients and physicians who have access to and are using secure messaging. This information can be used to target interventions to promote adoption and use of secure messaging.

## Introduction

Conceptually, expanding patient and clinician communications beyond health care facility walls improves patients’ access to care by providing a forum for patients to get answers to their questions without requiring in-person visits [1,2]. One mechanism to expand communication is through forms of computer-mediated communication such as email. Although early mechanisms to exchange email between patient and clinician were less secure, it is now common for email exchange to be conducted using a secure patient portal. Dubbed “secure messaging,” this form of communication is defined by the Centers for Medicare & Medicaid Services as “any electronic communication between a provider and patient that ensures only those parties can access the communication. This electronic message could be email or the electronic messaging function of a personal health record, an online patient portal, or any other electronic means” [2].

In 2001, the Institute of Medicine (IOM) noted that patient-provider communication via email had the potential to reduce costs while meeting patient needs more quickly [1]. Both patients and clinicians identified benefits of secure messaging, which included convenience, not feeling rushed, improved patient access, more direct and focused communication, increased efficiency, avoidance of phone tag, improved communication between visits, and improved patient engagement, satisfaction, and trust [3-7].

Since the publication of the IOM report, most physician practices have adopted secure messaging functionality, although these studies measured the number of physicians rather than the proportion of patients who have access to the functionality via their physicians [8-11]. Patients expressed interest in sending messages to their clinicians if given the opportunity, and they were receptive to receiving and reading the messages sent to them: the vast majority of messages sent to patients were read within 3 days, and fewer than 5% were not read within 3 weeks [12]. Yet a study by Tarver et al [13] estimated that only 3 in 10 individuals reported communicating with their clinicians using email or the internet in 2013.

In 2014, the Medicare and Medicaid electronic health record (EHR) Incentive Programs required that eligible professionals participating in stage 2 of the program use secure messaging to communicate with their patient population (not just Medicare beneficiaries) [2]. To receive incentive payments and avoid penalties, participating professionals had to use EHR systems that met meaningful use criteria defined by the Department of Health and Human Services (ie, certified health information technology [IT]). We would therefore expect that availability of the EHR functionality supporting secure messaging would increase as the 2014 requirement approached and continue to increase as more providers met the stage 2 criteria, and with similar requirements that were included in the first 2 years of the subsequent program (the Medicare Access and CHIP Reauthorization Act of 2015 [MACRA]).

Clinicians’ patterns of secure message communication affect patients’ use of the functionality: patients were more likely to initiate messages if their clinicians responded quickly and had a higher overall response rate [14]. Patients whose clinicians initiated more message threads were also more likely to initiate their own threads. We would therefore expect that patients’ use of secure messaging would increase as their access to that functionality via their providers’ infrastructure increased.

We used data from two nationally representative surveys to explore whether patients’ use of email exchange paralleled the availability of secure messaging functionality in ambulatory care settings. We present data pertaining to patients’ self-reported use of email to communicate with their clinicians and patients’ potential access to that functionality through physician visits.

## Methods

### Overview

Data from two nationally representative surveys were analyzed. In this section, the methodology associated with each survey is reported separately, starting first with methodologic approaches using data from the National Ambulatory Medical Care Survey (NAMCS), with which the proportion of office visits with access to secure messaging functionality were estimated. Approaches to using data from the National Health Interview Survey (NHIS) helped estimate the prevalence of patients who exchanged email with clinicians.

### National Ambulatory Medical Care Survey

The NAMCS is conducted annually by the National Center for Health Statistics (NCHS) using a nationally representative sample of ambulatory care medical visits to nonfederally employed office-based physicians. The NAMCS is an annual cross-sectional survey designed to provide estimates of ambulatory care medical visits to office-based physicians in the United States [15-19]. The survey captures information at both the individual physician and patient visit levels. Beginning in 2012, the NAMCS asked physicians if they regularly used, or had the functionality to send secure electronic messages (eg, email) to their patients, and in 2013, physicians were asked if they used certified health IT. We therefore analyzed NAMCS data from 2013 through 2016 to develop yearly snapshots of the availability of secure messaging to physicians and patients in the context of the ambulatory care medical visits.

#### National Ambulatory Medical Care Survey Study Population

The unit of measurement for the NAMCS was the patient-physician visit, excluding telephone consults, hospital visits, house calls, institutional settings, other visits performed outside the physician’s office, and visits made solely for administrative purposes (eg, leaving a specimen, paying a bill) [15,20].

NAMCS data were collected from physicians included in master lists from the American Medical Association and American Osteopathic Association who met the following criteria: office-based as defined by the respective association, principally engaged in patient care activities, and less than 85 years at the time of survey [15-19]. Physicians who were primarily employed in federal institutions or who had specialties of anesthesiology, pathology, or radiology were excluded. Each physician who met the criteria was assigned a random week during the year; if the physician saw no patients during that time period (eg, due to vacation or illness), he or she was excluded from the sample. In addition, the physician was excluded if patient visit data for the assigned time period was not recorded. Therefore, NAMCS data should not be considered representative of all ambulatory care office-based physicians. In our analyses, we used the prevalence of visits based on the physicians’ self-reported characteristics because this leverages the survey data as designed; it also allows us to better approximate the availability of secure messaging to patients with visits to physicians who can offer that functionality to communicate between visits.

Physician sampling was stratified based on census region, state, doctor type (Doctor of Medicine or Doctor of Osteopathy), practice type, metropolitan statistical area, and 14 specialty categories (Multimedia Appendix 1) [15]. Table 1 displays the number of respondents and unweighted response rates for the years included in our analyses.

**Table 1:** National Ambulatory Medical Care Survey response rates and number of sampled office visits, 2013-2016.

Survey year	Eligible physician respondents (unweighted response rate^a^), n (%)	Ambulatory visits, n
2016	721 (32.37)	13,165
2015	1415 (28.82)	28,332
2014	2325 (38.64)	45,710
2013	2879 (41.13)	54,873

^a^Conditional response rates reported for families, sample adults, and children; total response rate reported for households. Data in tables based on author’s compilation of information from data from the appropriate survey description documents published by the National Center for Health Statistics [15-17,19,21].

#### Dependent Variable From the National Ambulatory Medical Care Survey

Our dependent variable in NAMCS-related analyses was whether the physician conducting the ambulatory visit reported access to secure messaging. The question in the NAMCS was not specific to the visit itself; rather, it was related to the physician’s access to and use of (for surveys in 2013 through 2015) secure messaging functionality. This survey question changed with the 2016 survey to a dichotomous variable related to access to the secure messaging functionality, which is how we present the data in our analyses.

#### Independent Variables From the National Ambulatory Medical Care Survey

Our NAMCS-based analyses included both physician and practice characteristics. We included only one physician-level characteristic: physician specialty was identified based on data provided to the NCHS by the American Medical Association and the American Osteopathic Association [15]. We opted to use the NAMCS-defined categories for clinical specialty: primary care, medical specialty, and surgical specialty.

Practice characteristics included the use of certified health IT and practice size, ownership, and geographic region. We measured the use of certified health IT as an affirmative response to whether the physician’s current information system met the meaningful use criteria defined by the Department of Health and Human Services, which refers to the health IT certification criteria that support the Medicare and Medicaid Electronic Health Record Incentive programs (eg, *meaningful use*). Physicians who did not have an electronic system were coded as having no access to secure messaging.

Practice size and ownership were based on the practice in which each visit was conducted. We used a dichotomous practice size variable (solo/nonsolo). We included three categories for practice ownership: physician or physician group; insurance company or health plan; and academic medical center, community health center, or other hospital.

The geographic region was based on the location of the office in which the visit occurred and was categorized into census regions: Midwest, Northeast, South, and West. For all variables, nonresponse and unknown values were coded as missing.

### National Health Interview Survey

The NHIS, which was the second survey we analyzed for this research, is conducted annually by the NCHS using a nationally representative sample of civilian US residents. Beginning in 2011, the NHIS included a question asking adults if they communicated with their clinicians via email. Pairing responses to this question with sociodemographic information gathered through the NHIS provides a more comprehensive view of those in the United States who reported communicating with their clinicians using email. We included survey data only from 2013 through 2018 because the *internet use* variable was added in 2013, and internet access is a critical factor in patients’ ability to access secure messaging.

Similar to the NAMCS, the NHIS is an annual cross-sectional survey that uses a nationally representative sample of US residents selected based upon a complex, multistage-stratified sampling process [22,23]. The NHIS consists of four core components that capture information on the household, families within the sampled household, and a randomly selected child (when available) and adult from each family. Questions in the survey’s core components were asked consistently across survey years; Multimedia Appendix 1 lists the survey questions used for this study.

### National Health Interview Survey Study Population

NHIS study participants were randomly selected to represent the US population with oversampling for blacks, Asians, and adults older than 65 years within the sampled adults [23]. Excluded from the NHIS were individuals in long-term care and correctional facilities, as well as individuals living outside the country. Households with members of the Armed Forces were included only if at least one member in the family was not in the Armed Forces, in which case results only from the household members not in the Armed Forces were used in the final analyses.

Sampling was based on households, with approximately 36,000 households targeted each year to achieve a survey goal of about 87,500 individuals each year. In-person interviews were conducted with adults (individuals older than 17 years) in each household; this representative provided information about all members of the household. Each household was further subdivided into families with a responsible adult respondent for each family, and sampled children and adults (one of each per family). For each family sampled by NHIS, a sample adult was randomly selected to complete the *sample adult* questionnaire. If that sampled adult was absent at the time of the interview, the responsible adult family member could provide answers for them. We based our analyses on these sampled adults. Table 2 displays the survey response rate for each of the survey years we included in our analyses.

**Table 2:** Number of households, families, and adults included in each National Health Interview Survey, 2013-2017.

Respondent type	Survey (response rate^a^)					
	2018, n (%)	2017, n (%)	2016, n (%)	2015, n (%)	2014, n (%)	2013, n (%)
Households	29,839 (64.16)	32,617 (66.47)	40,220 (67.90)	41,493 (70.12)	44,552 (73.83)	41,335 (75.69)
Families	30,309 (98.73)	33,157 (98.90)	40,875 (98.85)	42,288 (98.89)	45,497 (98.98)	42,321 (98.96)
Sample adults	25,417 (83.89)	26,742 (80.69)	33,028 (80.86)	33,672 (79.66)	36,697 (80.54)	34,557 (81.71)

^a^Conditional response rates reported for families, sample adults, and children; total response rate reported for households. Data in tables based on author’s compilation of information from data from the appropriate survey description documents published by the National Center for Health Statistics [22,24-30].

#### Dependent Variable From National Health Interview Survey

Our dependent variable for NHIS-based analyses was individuals’ self-reported email communication with clinicians. The NHIS captures this element with five options: Yes, No, Refused, Not ascertained, and Don’t know. We created a dichotomous variable (yes, no) to capture this response and excluded individuals from analyses if their response was Refused, Not ascertained, or Don’t know.

#### Independent Variables From National Health Interview Survey

For NHIS analyses, we included age as a categorical variable (18-44 years, 45-64 years, and 65 years and older), sex, race (white, African American/black, and other race), Hispanic (yes, no), and census region (Northeast, Midwest, South, and West). On the basis of the literature about patient factors relevant to patient-centered communication that promotes improved outcomes [31,32], we included the variables for education and familiarity in speaking the English language in our analyses. Our education variable included six categories: less than a high school education, some college education but no degree, associate’s degree, bachelor’s degree, and graduate or professional degree. To assess individuals’ comfort with the English language, which is particularly relevant for written communication and patients’ comfort level when communicating with clinic staff [33,34], we used the responses to the NHIS question How well is English spoken, which included four categories (very well, well, not well, and not at all).

We also included a variable as a proxy for health care access. Our health insurance variable included five categories for private insurance, Medicare, Medicaid or other public insurance, military insurance, and uninsured. In addition, because we presented these data against those from a physician-based survey, we included a variable that assessed whether the individual saw or spoke with clinic staff in the 12 months preceding the survey. We created this as a categorical variable that distinguished between contact with a physician and contact with other clinician types. We created the physician’s designation by consolidating three NHIS questions about whether the patient saw a doctor who specializes in women’s health, a medical doctor who specializes in a particular medical disease or problem, and a general doctor who treats a variety of illnesses. The category for other clinician type included nurse practitioners, midwives, physician assistants, therapists, chiropractors, podiatrists, optometrists, ophthalmologists, or mental health professionals.

The ability to communicate with clinic staff using email depends on individuals’ access to and use of the internet. We therefore included a dichotomous variable to assess individuals’ use of the internet. Finally, we included a categorical variable to account for the survey year, one for each of the 6 years included in the analyses (2013 through 2018).

For all variables, unknown and nonresponses were coded as missing and those individuals excluded from the analyses.

### Statistical Analyses for Both Surveys

Our first regression model used NAMCS data, including survey year (2013 through 2016), practice characteristics (solo practice, practice ownership, and use of certified health IT), and physician specialty. The dependent variable for these analyses was the physician’s reported use of secure messaging. Our second regression model—based on NHIS data—included individuals’ characteristics (such as age, sex, race, ethnicity, geographic region, education, English language, and health insurance type), internet use, whether they saw or spoke with a clinician in the year preceding the survey, and survey year (2013 through 2018). The dependent variable for the NHIS analyses was self-reported email communication with physicians.

All analyses accounted for the complex sampling techniques used by NCHS by leveraging sample weights for stratification and primary sampling units. We estimated unadjusted statistical differences by year and individuals’ or physicians’ characteristics using chi-square test. We performed logistic regression to estimate associations between characteristics and the use of email or secure messaging. We used casewise deletion for all missing values (see Multimedia Appendix 2 for tables of missing values for each survey). Analyses were conducted using SAS software version 9.4 (SAS Institute Inc, Cary, NC).

## Results

### Email Availability and Use Across Both Surveys

**Figure 1:**
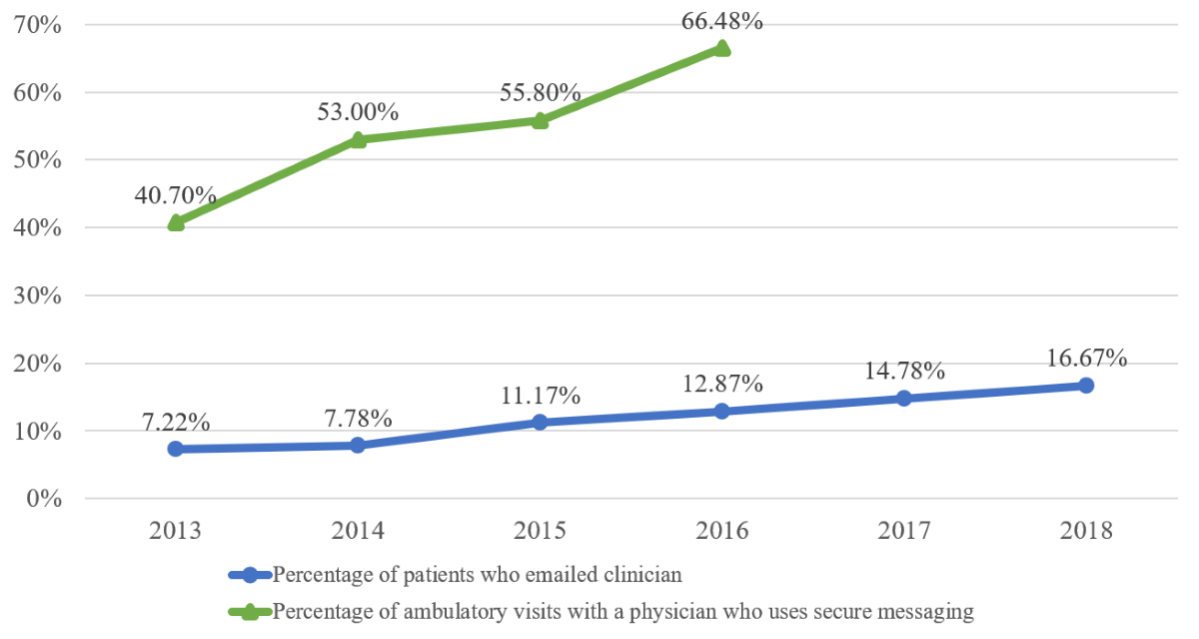
Prevalence of email use and access among US residents and ambulatory care visits, 2013-2018 (on the basis of authors’ analysis of National Center for Health Statistics [NCHS] and National Health Interview Surveys [NHIS], 2013-2018 [patients who emailed their clinician] and NCHS National Ambulatory Medical Care Surveys, 2013-2016 [ambulatory visits with physicians who used secure messaging]). Percentages are weighted national estimates.

Figure 1 displays prevalence data of email use by patients and physicians from both NHIS and NAMCS. Between 2013 and 2016, the percentage of ambulatory care visits with a physician who reported using secure messaging with patients increased 63% for a high of 66.48% in 2016. Although there was a larger percent increase (132%) between 2013 and 2018 of patients reporting the use of email to communicate with their clinicians, by 2018 only 16.67% of US residents reported using email to communicate with their clinicians.

### Physician and Practice Characteristics (National Ambulatory Medical Care Survey)

**Figure 2:**
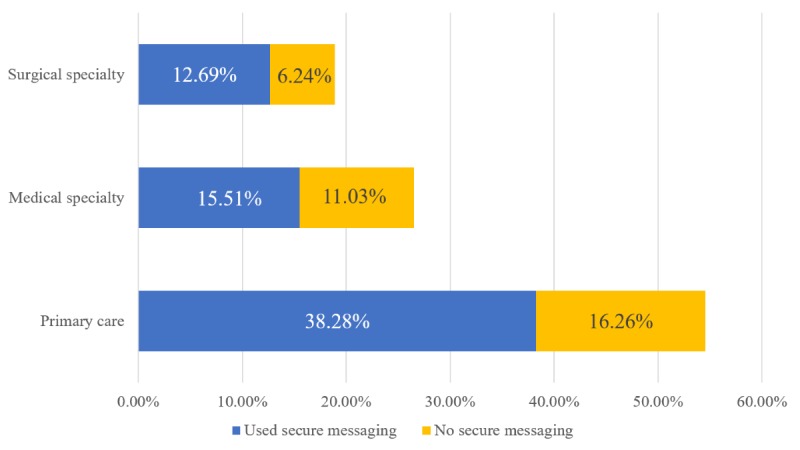
Percentage of ambulatory care visits by physicians’ specialty and secure messaging use, 2016 (on the basis of authors’ analysis of National Center for Health Statistics and National Ambulatory Medical Care Survey, 2015). Percentages are weighted national estimates of ambulatory care visits.

Figure 2 provides the percentages of ambulatory visits by physician specialty and use of secure messaging in 2016. A total of 70.19% of ambulatory medical care visits were conducted by primary care physicians who used secure messaging (38.28% of all visits). Two-thirds of surgical specialist visits were conducted by surgical specialists who used secure messaging (12.69% of all visits), while fewer than six in 10 medical specialist visits (15.51% of all visits) were conducted by those specialists who used secure messaging.

**Figure 3:**
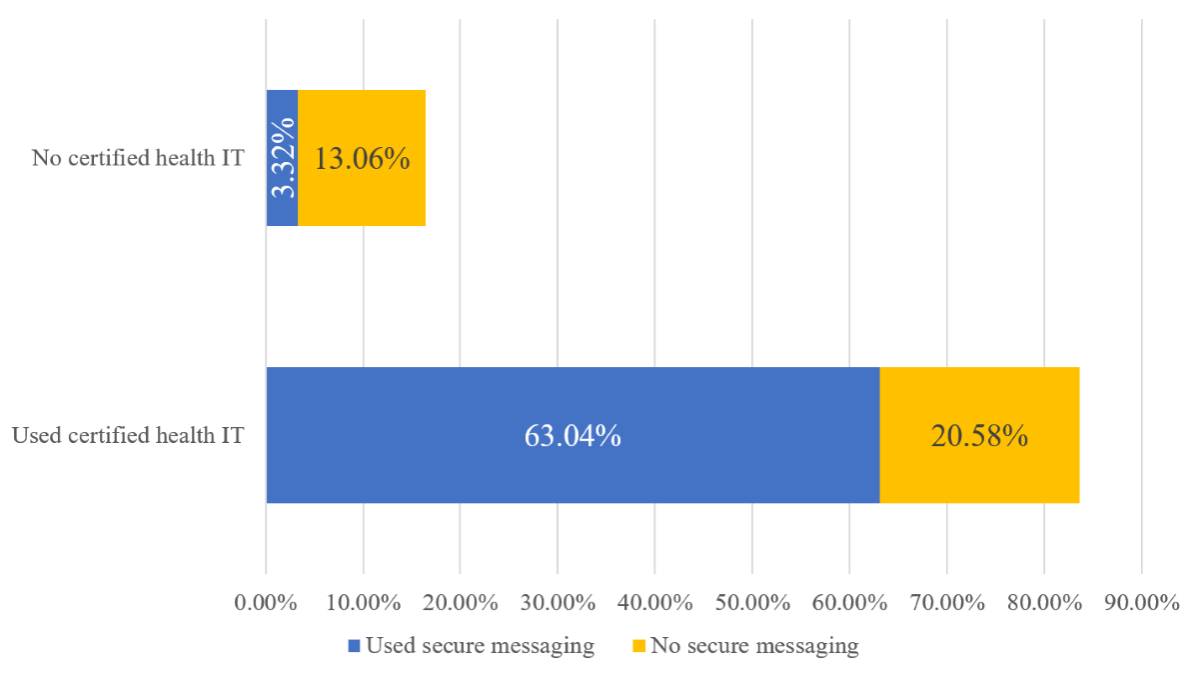
Percentage of ambulatory care visits by physicians’ use of certified health IT products and secure messaging use, 2016 (on the basis of authors’ analysis of National Center for Health Statistics and National Ambulatory Medical Care Survey, 2016). Percentages are weighted national estimates of ambulatory care visits.

Figure 3 displays the proportion of ambulatory care visits stratified by physicians’ use of certified health IT products and secure messaging use. A total of 83.62% of physician visits were conducted using certified health IT products, and three-quarters of those (or 63.04% of all visits) were conducted by physicians who reported secure messaging use. Of the 16.38% of ambulatory medical visits conducted by physicians not using certified health IT, only about 2 in 10 (or 3.32% of all visits) were performed by physicians using secure messaging.

**Figure 4:**
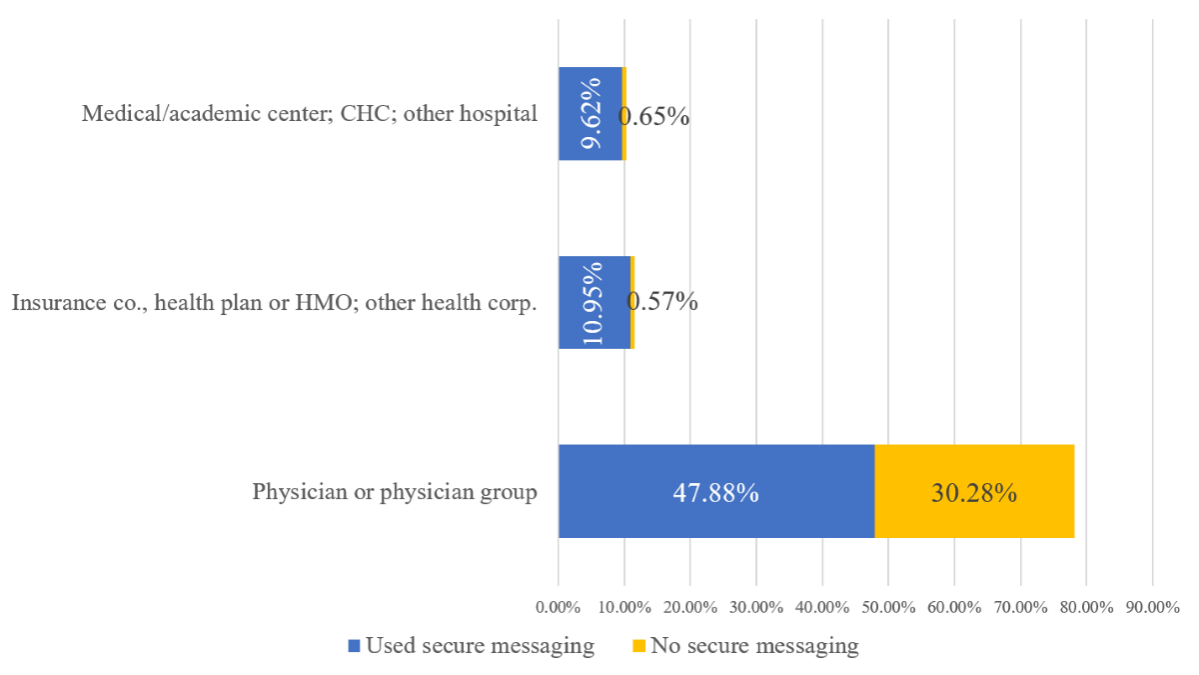
Percentage of ambulatory care visits by practice ownership and secure messaging use, 2016 (on the basis of authors’ analysis of National Center for Health Statistics and National Ambulatory Medical Care Survey, 2016). Percentages are weighted national estimates of ambulatory care visits. CHC: community health center; HMO: Health Maintenance Organization.

Figure 4 displays the percentages of ambulatory medical care visits conducted by physicians using secure messaging based on practice ownership. More than 90% of all ambulatory visits conducted in health maintenance organization (HMO) or insurance-owned practices and academic medical centers or hospital-owned organizations were conducted by physicians who used secure messaging. In contrast, slightly less than two-thirds of all visits at physician-owned practices were conducted by physicians who used secure messaging (47.88% of all ambulatory medical care visits).

**Figure 5:**
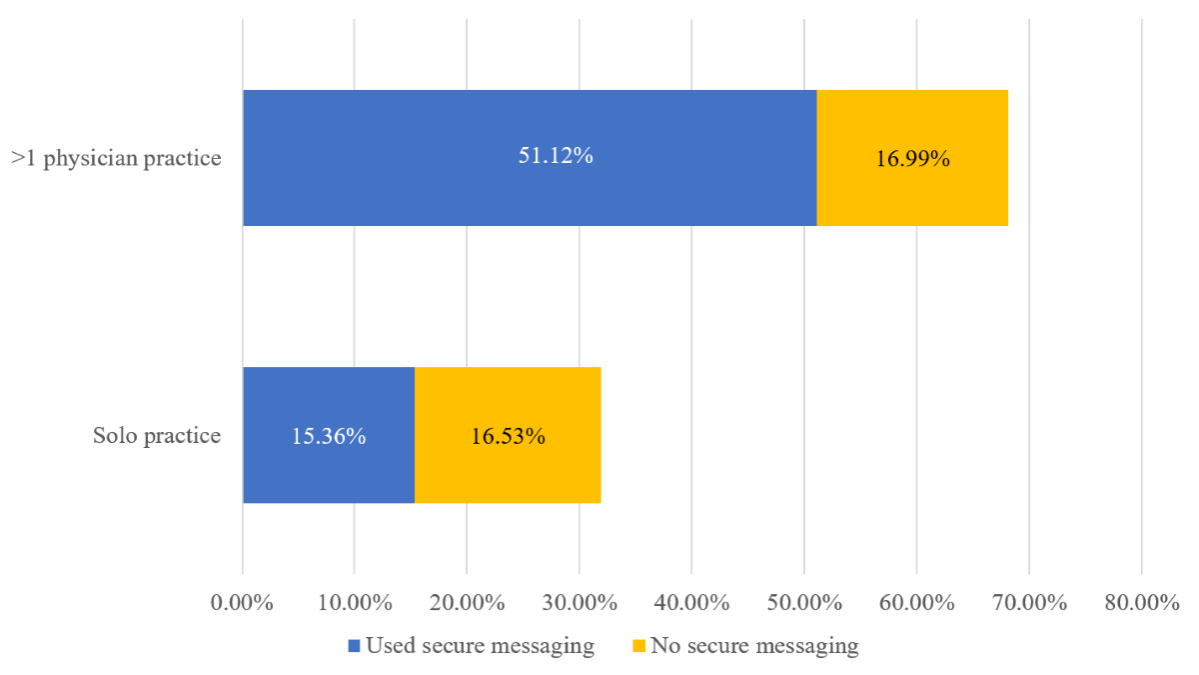
Percentage of ambulatory care visits by practice size and physicians’ reported secure messaging use, 2016 (on the basis of authors’ analysis of National Center for Health Statistics and National Ambulatory Medical Care Survey, 2016). Percentages are weighted national estimates of ambulatory care visits.).

Figure 5 displays the percentage of secure messaging use based on practice size. Slightly less than half of ambulatory care visits in solo physician practices (15.36% of all visits) were conducted by physicians who had access to secure messaging functionality. In contrast, three-quarters of ambulatory visits in practices with more than 1 physician were conducted by physicians who used secure messaging (51.12% of all visits).

Multimedia Appendix 3 presents a comparison between 2013 and 2016 of the prevalence of secure messaging use by physician and practice characteristics. Across all characteristics, there was a statistically significant increase over the 4 years, which persisted in adjusted analyses. Table 3 presents the adjusted estimates of association, controlling for physician and practice characteristics. No differences were observed by specialty. Physicians using certified health IT had greater odds of using secure messaging (OR 11.46, 95% CI 7.55-17.39) than physicians not using certified products. Ambulatory care visits in solo physician practices had lower odds of being conducted by physicians who used secure messaging (OR 0.46, 95% CI 0.35-0.60) than those in practices with more than 1 physician. Visits conducted in physician-owned practices had lower odds of being conducted by physicians using secure messaging than practices owned by other entities.

**Table 3:** Regression results for association between ambulatory care with secure messaging functionality and physician and practice characteristics, based on authors’ analysis of National Center for Health Statistics, National Ambulatory Medical Care Surveys, 2013-2016.

Characteristic and independent variables			OR^a^ (95% CI)		*P* value
**Certified health IT^b^ use**					
	Yes	11.46 (7.55-17.39)		<.001	
	No	Referent		Referent	
**Solo physician practice**					
	Yes	0.46 (0.35-0.60)		<.001	
	No	Referent		Referent	
**Practice ownership**					
	Insurance company, health plan, or HMO^c^; other health corporation	1.81 (1.29-2.54)		<.001	
	Medical/academic health center; CHC^d^; other hospital	2.12 (1.57-2.87)		<.001	
	Physician or physician group	Referent		Referent	
**Physician specialty**					
	Medical	0.83 (0.62-1.11)		.20	
	Surgical	0.90 (0.70-1.16)		.42	
	Primary care	Referent		Referent	
**Region**					
	Midwest	0.85 (0.61-1.18)		.32	
	Northeast	0.55 (0.38-0.80)		<.01	
	South	0.94 (0.66-1.34)		.74	
	West	Referent		Referent	
**Survey year**					
	2013	0.29 (0.21-0.42)		<.001	
	2014	0.55 (0.38-0.79)		<.01	
	2015	0.60 (0.40-0.90)		.01	
	2016	Referent		Referent	

^a^OR: odds ratio.

^b^IT: information technology.

^c^HMO: health maintenance organization.

^d^CHC: community health center.

### Patient Characteristics (National Health Interview Survey)

Table 4 lists the 2013 and 2018 reported rates of email communication with clinicians by individuals’ sociodemographic characteristics. Prevalence of email communication increased across all categories except among individuals who did not speak English well or at all. We observed large percent increases across the 6 years among individuals with Medicare (236% change from 4.05% to 13.63%) and military insurance (154% change from 9.06% to 23.03%), and those who were uninsured (146% change from 1.91% to 4.71%). We also found a large increase in communication via email among Hispanic individuals (180% change from 3.36% to 9.41%). By education level, the largest percent change was among individuals with a high school diploma or its equivalent (165% change from 3.26% to 8.66%).

**Table 4:** Prevalence of email communication with clinicians by individuals’ characteristics based on authors’ analysis of National Center for Health Statistics, National Health Interview Surveys, 2013 and 2018. Percentages are weighted national estimates.

Characteristics		Individuals emailing clinicians in 2013, % (95% CI)	Individuals emailing clinicians in 2018, % (95% CI)	*P* value	
**Age (years)**					**<.001**
	18-44	6.82 (6.25-7.39)	15.98 (14.87-17.10)		
	45-64	8.81 (8.12-9.50)	19.15 (17.97-20.33)		
	65+	5.23 (4.47-6.00)	14.21 (13.09-15.32)		
**Sex**					**<.001**
	Male	6.02 (5.47-6.57)	14.25 (13.29-15.22)		
	Female	8.33 (7.77-8.89)	18.93 (17.83-20.02)		
**Education**					**<.001**
	Less than a high school diploma	1.00 (0.65-1.36)	2.55 (1.84-3.26)		
	High school diploma or equivalent	3.26 (2.74-3.79)	8.66 (7.74-9.58)		
	Some college, no degree	6.54 (5.69-7.39)	15.21 (13.74-16.68)		
	Associate’s degree	6.97 (5.92-8.01)	17.41 (15.72-19.09)		
	Bachelor’s degree	12.87 (11.87-13.86)	25.18 (23.51-26.86)		
	Graduate or professional degree	17.06 (15.49-18.64)	33.23 (31.03-35.43)		
**Hispanic**					**<.001**
	Yes	3.36 (2.78-3.93)	9.41 (8.00-10.83)		
	No	7.91 (7.50-8.31)	18.09 (17.23-18.95)		
**Race**					**<.001**
	Black	4.68 (3.85-5.50)	11.07 (9.74-12.40)		
	Other race	9.07 (7.71-10.43)	16.86 (14.17-19.56)		
	White	7.45 (7.04-7.86)	17.56 (16.64-18.48)		
**Speak English**					
	Not at all	0.30 (0.00-0.77)	0.48 (0.00-1.15)	.67	
	Not well	1.13 (0.58-1.68)	0.75 (0.16-1.34)	.35	
	Well	3.51 (2.49-4.54)	9.48 (7.40-11.55)	<.001	
	Very well	8.39 (7.75-9.03)	18.16 (17.29-19.04)	<.001	
**Region**					**<.001**
	Northeast	6.35 (5.57-7.14)	15.59 (13.77-17.42)		
	Midwest	6.05 (5.25-6.84)	16.28 (14.78-17.78)		
	South	5.88 (5.37-6.40)	14.54 (13.28-15.80)		
	West	11.17 (10.29-12.06)	21.10 (18.78-23.42)		
**Health insurance**					**<.001**
	Uninsured	1.91 (1.48-2.34)	4.71 (3.70-5.73)		
	Medicaid or other public payer	3.76 (2.90-4.62)	7.70 (6.48-8.92)		
	Medicare	4.05 (3.32-4.78)	13.63 (12.14-15.12)		
	Military	9.06 (6.92-11.19)	23.03 (18.35-27.72)		
	Private	9.61 (9.08-10.13)	20.54 (19.50-21.57)		
**Saw/spoke to clinician in prior 12 months**					**<.001**
	Physician	8.81 (8.36-9.27)	20.01 (19.03-20.99)		
	Nonphysician clinician	4.85 (3.65-6.04)	9.23 (7.53-10.93)		
	None	1.54 (1.1-1.98)	3.34 (2.54-4.14)		
**Internet use**					**<.001**
	Yes	9.38 (8.93-9.84)	20.09 (19.13-21.04)		
	No	0.44 (0.23-0.65)	1.28 (0.85-1.71)		

In 2018, the highest percentage of individuals using email to communicate with their clinicians lived in the West (21.10%), saw or spoke with their physician in the preceding 12 months (20.01%), and were between the ages of 45 and 64 years (19.96%). Almost twice as many non-Hispanics reported email communication compared with Hispanics (18.09% vs 9.41%). Fewer black individuals (11.07%) reported using email to communicate with their clinicians compared with white individuals (17.56%).

Table 5 lists multivariate analysis results using NHIS data from 2013 through 2018. Individuals with a graduate or professional degree had greater odds of communicating with clinicians using email than individuals with less education. The English language was also positively associated with email communication, with individuals who spoke English very well having greater odds for email communication with clinicians than individuals who English language skills were less well developed. After controlling for other variables in the model, we found no statistical difference between the youngest individuals and oldest individuals (*P*=.57), nor between white individuals and those of other races (*P*=.73). Black race was associated with reduced odds of communicating using email (OR 0.83, 95% CI 0.77-0.90). Hispanic individuals had lower odds for communicating with clinicians using email than non-Hispanic individuals (OR 0.79, 95% CI 0.73-0.86).

We observed large odds for email use among individuals who saw or spoke with a physician (OR 4.81, 95% CI 4.31-5.36) or nonphysician clinician (OR 2.19, 95% CI 1.89-2.53) in the preceding year. Uninsured individuals, and those with Medicaid or other public payers, had lower odds of communicating via email with clinicians than individuals with private payers; however, we found no statistical difference between individuals with private payers and individuals with either military insurance or Medicare.

Differences by region mirrored what we observed in the regional differences from the NAMCS analyses. As expected, internet use was strongly associated with email communication with clinicians (OR 11.10, 95% CI 9.39-13.11).

**Table 5:** Adjusted associations between individuals’ characteristics and email communication with clinicians based on authors’ analysis of National Center for Health Statistics, National Health Interview Surveys, 2013-2018.

Independent variable		OR^a^ (95% CI)	*P* value
**Age (years)**			
	18-44	1.02 (0.95-1.09)	.57
	45-64	1.24 (1.16-1.32)	<.001
	65 years and older	Referent	Referent
**Sex**			
	Female	0.80 (0.77-0.84)	<.001
	Male	Referent	Referent
**Education**			
	Less than a high school diploma	0.18 (0.16-0.21)	<.001
	High school diploma or equivalent	0.29 (0.27-0.31)	<.001
	Some college, no degree	0.43 (0.40-0.47)	<.001
	Associate’s degree	0.46 (0.43-0.50)	<.001
	Bachelor’s degree	0.71 (0.67-0.76)	<.001
	Graduate or professional degree	Referent	Referent
**Hispanic**			
	Yes	0.79 (0.73-0.86)	<.001
	No	Referent	Referent
**Race**			
	Black	0.83 (0.77-0.90)	<.001
	Other race	0.98 (0.90-1.08)	.73
	White	Referent	Referent
**Speak English**			
	Not at all	0.29 (0.17-0.51)	<.001
	Not well	0.30 (0.22-0.41)	<.001
	Well	0.73 (0.64-0.84)	<.001
	Very well	Referent	Referent
**Region**			
	Northeast	0.50 (0.45-0.57)	<.001
	Midwest	0.62 (0.55-0.68)	<.001
	South	0.58 (0.52-0.64)	<.001
	West	Referent	Referent
**Health insurance**			
	Uninsured	0.52 (0.46-0.58)	<.001
	Medicaid or other public payer	0.65 (0.59-0.71)	<.001
	Medicare	1.01 (0.93-1.09)	.88
	Military	1.11 (0.97-1.28)	.13
	Private	Referent	Referent
**Saw/spoke to clinician in prior 12 months**			
	Physician	4.81 (4.31-5.36)	<.001
	Nonphysician clinician	2.19 (1.89-2.53)	<.001
	None	Referent	Referent
**Internet use**			
	Yes	11.10 (9.39-13.11)	<.001
	No	Referent	Referent
**Survey year**			
	2013	0.43 (0.39-0.48)	<.001
	2014	0.42 (0.39-0.46)	<.001
	2015	0.63 (0.59-0.69)	<.001
	2016	0.74 (0.69-0.79)	<.001
	2017	0.86 (0.80-0.92)	<.001
	2018	Referent	Referent

^a^OR: odds ratio.

## Discussion

### Principal Findings

This research leverages data from two large nationally representative surveys to provide longitudinal prevalence rates for the use of, and access to, email to communicate with clinicians. Between 2013 and 2016, we observed a statistically significant increase in ambulatory medical care visits conducted by physicians who used secure messaging, such that by 2016, two-thirds of all visits were administered by physicians who used secure messaging. We observed significant positive associations between certified health IT use and secure messaging use.

The strong association of secure messaging use with certified health IT may be attributed to the requirements of the meaningful use program, and its subsequent replacement program (MACRA), for eligible providers to report on secure messaging use. Because it takes time to implement workflows to support IT changes, the increase between 2013 and 2014 demonstrated in this study may be associated with physicians increasing their access and use of secure messaging in anticipation of the stage 2 requirements. Moreover, visits conducted by physicians without certified health IT had lower odds of having secure messaging functionality. Although our data do not provide a cause-and-effect answer, the timing and degree of the increases give credence to the idea that much of the uptake by physicians was due to meaningful use and MACRA. Because secure messaging has shown to be positively associated with patient health outcomes [35-42], driving physicians’ behavior through similar programs and regulations may be important for policy makers to consider as they seek ways to increase patient engagement and promote improved health outcomes.

In contrast to the two-thirds ambulatory medical care visits with secure messaging access in 2016, 2 years later in 2018, only 17% of US residents reported communicating with their clinicians using email. Individuals who saw or spoke with a physician in the preceding year had greater odds of communicating via email with a clinician during that time period. Clinicians’ secure messaging use has been demonstrated to influence patients’ messaging behaviors [14,43]. Our findings demonstrate that most physicians have secure messaging capabilities, but patients were not taking advantage to communicate using that modality with this clinic staff. It seems that access to secure messaging functionality alone may be insufficient to change behaviors.

Many patients expressed intention to send messages to their clinicians if given the opportunity [5,33]. In addition, patients seemed receptive to receiving and reading the messages sent to them: the vast majority of messages sent to patients were read within 3 days, and fewer than 5% of messages were not read within 3 weeks [12]. Therefore, there may be a need for physicians to encourage patients to use the secure messaging forum to communicate to if we wish to see an increase in patients’ use of secure messaging.

There is moderate supporting evidence of associations between message use and selected patient outcomes (eg, glucose levels in patients with diabetes), and some evidence for other outcomes (eg, diastolic and systolic blood pressure among patients with hypertension) [44]. We observed significant differences in email communication by individuals based on education, race, ethnicity, and insurance status, with patients with lower levels of education, black patients, those with Medicaid or other public payers, and uninsured patients having reduced odds for secure messaging use. Such differences in the use of a communication modality that might have positive impacts on health outcomes—which permits patients to communicate with clinic staff at their convenience and can increase satisfaction and improve understanding of their condition [6,45]—may further exacerbate health disparities if not addressed.

The discrepancies in the proportion of ambulatory care visits associated with access to and use of secure messaging use by practice size and ownership may be due to limited staffing resources of solo and physician-owned practices. Clinical responses to patient-generated messages were frequently triaged through a clinical response team that might include nurses (registered, licensed practical, or advanced practice), physician assistants, pharmacists, and physicians [46-48]. Effective workflow design may be critical to gaining acceptance of secure messaging among clinical teams because workflows facilitating this team-based approach to response may be complicated and confusing [48]. Development of strategies and resources that are effective for less well-resourced physician practices may be critical to promoting secure messaging use among patients.

### Limitations

The NHIS question asked about individuals’ email exchange with clinicians and did not specifically mention secure messaging. Therefore, it is possible that the estimates provided in this study may overestimate secure messaging use among patients. If the NHIS had asked respondents about secure messaging use, it is likely that many would be unfamiliar with the phrase and may not have responded accurately, resulting in underestimation of messaging use. Most email communication is facilitated through patient portals as secure messages, so the estimates presented in this paper around email exchange are likely to be a close approximation of the use of secure messaging.

Not surprisingly, individuals who saw or spoke with a physician in the year preceding the survey had four times greater odds for using email to communicate with their clinicians than individuals who did not have an interaction with a clinician during that time. From these data, it is not clear if the email communication precipitated the clinical encounter or vice versa. There is no way to determine causality with cross-sectional surveys such as NHIS and NAMCS. There was also no information on whether there was bidirectional exchange using email (eg, patient wrote to clinician and received a response). Prior studies on the relationship between secure messaging and reduced health care utilization were mixed, and it is clear from these results that further research is warranted to better understand that relationship [10,11,39,40].

The outcomes of interest and many of the independent variables were based on self-report and therefore subject to recall bias. NCHS, however, uses validated questions that should reduce the effect of that bias.

#### Missing Data

In 2015, the NAMCS’ physician response rate was less than 30%, which could lead to a nonresponse bias. A detailed analysis of potential nonresponse bias conducted by NCHS in 2012 found no evidence of nonresponse bias based on census division, metropolitan practice location, and physician specialty [15]. However, NCHS did find evidence that the sample may overestimate solo practitioners and underestimate in large (11 physicians or greater) and HMO-owned practices. The differences we observed by practice ownership and size (which we proxied as solo vs nonsolo) were large, so even if there was a bias toward the null based on the nonresponse bias identified by NCHS, it is likely that the statistical differences would persist.

We found a larger percentage of missing data for our dependent variable (19%) in the 2014 NAMCS data compared with that in other years, which ranged between 2% and 3%. There is no information in the NAMCS documentation about this gap. It does raise a concern about the viability of the estimates for that survey year, and those data should be viewed with caution. The missing data rates for all other variables across the surveys were less than 5% (see Multimedia Appendix 2).

In contrast, the response rates for the NHIS were acceptable, with a household response rate ranging between 64% and 76%, and a sampled adult response rate of approximately around 80%. The NCHS conducts the survey in-person and uses flash cards to aid in the understanding of the questions. The random selection of the adult for that questionnaire is weighted toward blacks, Asians, and adults older than 65 years to ensure sufficient sample for those populations.

We found a 50% missing rate for the English language variable for 2013. Here again, survey documentation provided no indication about why there was such a difference in missing rates for 2013 (all subsequent years had less than 1% missing), but those data should be viewed with caution. Similarly, 8% of individuals had missing data for the internet use variable in 2015. All other NHIS variables had missing rates below 5%.

### Comparison With Prior Work

The overall percentage of almost 17% using email to communicate with their clinicians is lower than what would be expected, given the findings from other studies that indicated that many patients expressed intention to send messages to their clinicians if given the opportunity [5,13,33]. These data use a cross-section of US residents, which includes both healthy and sick individuals. In other studies, patients currently receiving clinical treatment reported few barriers to use, including challenges in accessing the patient portal to send or receive a message, doubts about the reliability of the messaging function or prior bad experiences, concern about imposing on clinicians’ time, and perceived resistance to use of messaging among clinical staff [3,49,50]. In addition, studies identified clinician behavior as a significant driver of secure messaging use among patients [14,43]. For example, patients were more likely to initiate their own messages if their clinicians responded quickly, had a higher overall response rate, or initiated their own messages [14]. Our analyses found no difference in access to the capability by physician specialty, even though prior research found most patients sent messages to their primary care clinicians [6]. Therefore, to promote adoption of email communication among patients, it may be important for clinicians to promote and advocate for its use.

Our findings of the characteristics associated with patients’ self-reported communication with clinicians were largely consistent with other research conducted at single sites or within large integrated delivery networks. Our study is the first to use a large, nationally representative sample as is available through the NHIS. Consistent with a number of studies [5,13,51,52], we found higher education to be positively associated with secure messaging use. Similar to our findings as well, several studies found privately insured patients were more likely to use secure messaging [13,36,41]. Only one study examined associations with primary language and secure messaging use [33], and their findings were also consistent with our findings on English language familiarity. Similar to our findings, most studies that examined the association by race found that white patients had higher rates of secure messaging use than other races [5,36,41,45,53-55].

After controlling for other characteristics in our multivariate models, we found that women had reduced odds of using email to communicate with clinicians. This is in contrast to a number of studies that found the reverse [5,13,36,41,45,53,55,56]. Our unadjusted numbers showed a higher percentage of female patients reporting email communication with clinicians than males; it was only after adjusting for other sociodemographic factors that we found the reduced odds. The difference between our research and those studies is that our adjusted analyses controlled for education, health insurance, and the English language.

### Conclusions

Aligning analyses of the NHIS and NAMCS presents a unique opportunity to understand patients’ potential access to and use of secure messaging over time. These analyses are novel because prior studies were based on data from individual health care organizations or integrated delivery networks, or used smaller national survey data that asked about email and internet communication with clinicians. There are no published data that compare secure messaging by practice ownership and size. This is the first study to use a large nationally representative sample to explore the association between selected social determinants of health and email exchange between patients and clinicians.

In 2009, Street et al [31] published a framework that describes how communication functions such as information sharing may directly and indirectly lead to improved patient outcomes. Many secure messaging benefits cited by clinicians—improvements in access, more direct and focused communication between patients and clinic staff, improved efficiency including avoidance of phone tag, improved communication between visits, and improved patient engagement, satisfaction, and trust [3,4]—are constructs in the Street et al [31] pathway between communication functions and improved patient outcomes. Unfortunately, our data demonstrate that there is unequal use of secure messaging among patients based on social and demographic characteristics, and that secure messaging use by patients is not increasing as the functionality is made available through office visits. Developing a better understanding of who is using secure messaging, and whether and how clinicians are encouraging that use, may permit better targeting of secure messaging interventions that encourage secure messaging adoption and use.

## Appendix

Multimedia Appendix 1 Selected National Health Interview Survey and National Ambulatory Medical Care Survey questions and responses included in analyses: lists the survey questions and response options included in the analyses.

Multimedia Appendix 2 Percentage of missing data by survey, variable, and year: provides 2 tables—1 for each survey—with the missing data rates by variable and year for all dependent and independent variables used in the analyses.

Multimedia Appendix 3 Prevalence (95% CI) of secure message use by physicians conducting ambulatory care medical visits. Table of the percentages (with 95% CI) of ambulatory medical care visits conducted by a physician who used secure messaging in 2013 and 2016, plus the P value to indicate statistical difference between the years.

## Abbreviations

EHR: electronic health record
HMO: Health Maintenance Organization
IOM: Institute of Medicine
IT: information technology
MACRA: Medicare Access and CHIP Reauthorization Act of 2015
NAMCS: National Ambulatory Medical Care Survey
NCHS: National Center for Health Statistics
NHIS: National Health Interview Survey
