# Supplementary material for: Disparities in Secure Messaging Uptake Between Patients and Physicians: Longitudinal Analysis of Two National Cross-Sectional Surveys
Source: J Med Internet Res. 2020 May 1;22(5):e12611. doi: 10.2196/12611 (PMC7229528; doi:10.2196/12611)
Supplement: Multimedia Appendix 1 [file jmir_v22i5e12611_app1.docx]

Multimedia Appendix 1.
Selected NHIS and NAMCS questions and responses included in analyses

| Survey | Survey Question | Response Options | Years Question was Included in Survey |
| --- | --- | --- | --- |
| NHIS | NCHS calculated based on respondents' birthdate | 00 Under 1 year 01-84 1-84 years 85 85+ years | 2013-2018 |
| NHIS | DURING THE PAST 12 MONTHS, have you seen or talked to any of the following health care providers about your own health? ..... A mental health professional such as a psychiatrist, psychologist, psychiatric nurse, or clinical social worker | 1 Yes 2 No 7 Refused 8 Not ascertained 9 Don't know | 2013-2018 |
| NHIS | DURING THE PAST 12 MONTHS, have you seen or talked to any of the following health care providers about your own health? ..... An optometrist, ophthalmologist, or eye doctor (someone who prescribes eyeglasses) | 1 Yes 2 No 7 Refused 8 Not ascertained 9 Don't know | 2013-2018 |
| NHIS | DURING THE PAST 12 MONTHS, have you seen or talked to any of the following health care providers about your own health? ..... A foot doctor | 1 Yes 2 No 7 Refused 8 Not ascertained 9 Don't know | 2013-2018 |
| NHIS | DURING THE PAST 12 MONTHS, have you seen or talked to any of the following health care providers about your own health? ..... A chiropractor | 1 Yes 2 No 7 Refused 8 Not ascertained 9 Don't know | 2013-2018 |
| NHIS | DURING THE PAST 12 MONTHS, have you seen or talked to any of the following health care providers about your own health? ..... A physical therapist, speech therapist, respiratory therapist, audiologist, or occupational therapist | 1 Yes 2 No 7 Refused 8 Not ascertained 9 Don't know | 2013-2018 |
| NHIS | DURING THE PAST 12 MONTHS, have you seen or talked to any of the following health care providers about your own health? ..... A nurse practitioner, physician assistant, or midwife | 1 Yes 2 No 7 Refused 8 Not ascertained 9 Don't know | 2013-2018 |
| NHIS | DURING THE PAST 12 MONTHS, have you seen or talked to any of the following health care providers about your own health? ..... A doctor who specializes in women's health (an obstetrician/gynecologist)? | 1 Yes 2 No 7 Refused 8 Not ascertained 9 Don't know | 2013-2018 |
| NHIS | DURING THE PAST 12 MONTHS, have you seen or talked to any of the following health care providers about your own health? ..... A medical doctor who specializes in a particular medical disease or problem (other than obstetrician/ gynecologist, psychiatrist or ophthalmologist)? | 1 Yes 2 No 7 Refused 8 Not ascertained 9 Don't know | 2013-2018 |
| NHIS | DURING THE PAST 12 MONTHS, have you seen or talked to any of the following health care providers about your own health? ..... A general doctor who treats a variety of illnesses (a doctor in general practice, family medicine, or internal medicine) | 1 Yes 2 No 7 Refused 8 Not ascertained 9 Don't know | 2013-2018 |
| NHIS | Please give me the number of the group that represents [your/ person's] Hispanic origin or ancestry | 00 Multiple Hispanic 01 Puerto Rico 02 Mexican 03 Mexican-American 04 Cuban/Cuban American 05 Dominican (Republic) 06 Central or South American 07 Other Latin American, type not specified 08 Other Spanish 09 Hispanic/Latino/Spanish, non-specific type 10 Hispanic/Latino/Spanish, type refused 11 Hispanic/Latino/Spanish, type not ascertained 12 Not Hispanic/Spanish origin | 2013-2018 |
| NHIS | DURING THE PAST 12 MONTHS, have you ever used computers for any of the following …Communicate with a health care provider by email | 1 Yes 2 No 7 Refused 8 Not ascertained 9 Don’t know | 2013-2018 |
| NHIS | Primary or main race reported by respondent | 01 White  02 Black/African American  03 Indian (American), Alaska Native  09 Asian Indian  10 Chinese  11 Filipino  15 Other Asian*  16 Primary race not releasable**  17 Multiple race, no primary race selected | 2013-2018 |
| NHIS | Data calculated by NCHS | 1 Northeast 2 Midwest 3 South 4 West | 2013-2018 |
| NHIS | Are you a male or female? | 1 Male 2 Female | 2013-2018 |
| NHIS | How well [fill: do you/ does ALIAS] speak English? | 1 Very well  2 Well  3 Not well  4 Not at all  7 Refused  9 Don’t know | 2013-2018 |
| NHIS | What is the HIGHEST level of school [fill: you have/ALIAS has] completed or the highest degree [fill: you have/ALIAS has] received? | 00 Never attended/kindergarten only 01 1st grade  02 2nd grade  03 3rd grade  04 4th grade  05 5th grade  06 6th grade  07 7th grade  08 8th grade  09 9th grade  10 10th grade  11 11th grade  12 12th grade, no diploma  13 GED or equivalent  14 High School Graduate  15 Some college, no degree 16 Associate degree: occupational, technical, or vocational program  17 Associate degree: academic program  18 Bachelor's degree (Example: BA, AB, BS, BBA)  19 Master's degree (Example: MA, MS, MEng, MEd, MBA)  20 Professional School degree (Example: MD, DDS, DVM, JD)  21 Doctoral degree (Example: PhD, EdD)  96 Child under 5 years old  97 Refused  99 Don't know | 2013-2018 |
| NHIS | Insurance status based on NHIS recoded variables: MEDICAID, MEDICARE, MILITARN (or MILTAR for years 2013-2017), NOTCOV, PRIVATE |  | 2013-2018 |
| NHIS | Do you use the Internet? | 1 Yes 2 No 7 Refused 9 Don’t know | 2013-2018 |
| NAMCS | Please indicate whether your practice has each of the following computerized capabilities and how often these capabilities are used:  Exchanging secure messages with patients | -9 = Blank  -8 = Don’t know  -6 = Refused to answer question  1 = Yes, used routinely  2 = Yes, but NOT used routinely  3 = Yes, but turned off or not used  4 = No | 2013-2015 |
| NAMCS | Please indicate whether your practice has each of the following computerized capabilities and how often these capabilities are used:  Exchanging secure messages with patients | -9 = Blank  -8 = Don’t know  -6 = Refused to answer question  1 = Yes  2 = No | 2016 |
| NAMCS | Does your current system meet meaningful use criteria as defined by the Department of Health and Human Services? | -9 = Blank  -8 = Don’t know  -6 = Refused to answer question  1 = Yes  2 = No | 2013-2016 |
| NAMCS | Physician specialty | 01 = General and family practice  03 = Internal medicine  04 = Pediatrics  05 = General surgery  06 = Obstetrics and gynecology  07 = Orthopedic surgery  08 = Cardiovascular diseases  09 = Dermatology  10 = Urology  11 = Psychiatry  12 = Neurology  13 = Ophthalmology  14 = Otolaryngology  15 = All other | 2013-2016 |
| NAMCS | Do you have a solo practice, or are you associated with other physicians in a partnership, a group practice, or some other way at this visit location? | -9 = Blank  -8 = Unknown  -6 = Refused to answer question  1 = Solo  2 = Non-solo | 2013-2016 |
| NAMCS | Who owns the practice at this visit location? | -9 = Blank  -8 = Unknown  -6 = Refused to answer question  1 = Physician or physician group  2 = Medical/Academic health center; other hospital  3 = Insurance company, health plan, or HMO; other health care Corporation; other | 2013-2016 |
| NAMCS | Data calculated by NCHS | 1 Northeast 2 Midwest 3 South 4 West | 2013-2016 |
|  |  |  |  |
