# Supplementary material for: Disparities in Secure Messaging Uptake Between Patients and Physicians: Longitudinal Analysis of Two National Cross-Sectional Surveys
Source: J Med Internet Res. 2020 May 1;22(5):e12611. doi: 10.2196/12611 (PMC7229528; doi:10.2196/12611)
Supplement: Multimedia Appendix 2 [file jmir_v22i5e12611_app2.docx]

Multimedia Appendix 2. Percentage of Missing Data by Survey, Variable, and Year

Appendix Table 2-1. Percentage of Missing Data for Variables Derived from the NAMCS^,a^

| Year | **2013** | **2014** | **2015** | **2016** |
| --- | --- | --- | --- | --- |
| Secure messaging use | 2.47 | 1.90 | 2.02 | 1.36 |
| Certified health IT | 2.80 | 19.46 | 1.94 | 1.75 |
| Practice ownership | 3.43 | 3.28 | 3.29 | 4.29 |
| Practice size (solo vs >1 physician) | 0.03 | 0.14 | 0.00 | 0.17 |

^a^ Based on authors’ analysis of NCHS, National Ambulatory Medical Care Surveys, 2013-2016

^b^ Percentages are weighted national estimates of ambulatory medical care visits in the United States. Physician specialty and region had no missing data.

Appendix Table 2-1. Percentage of Missing Data for Variables Derived from the NHIS^a, b^

| Year | **2013** | **2014** | **2015** | **2016** | **2017** | **2018** |
| --- | --- | --- | --- | --- | --- | --- |
| Education | 0.46 | 0.53 | 0.53 | 0.41 | 0.41 | 0.54 |
| Email | 1.42 | 1.52 | 1.73 | 1.41 | 1.29 | 1.16 |
| Health insurance | 0.40 | 0.52 | 0.63 | 0.62 | 0.50 | 0.51 |
| Internet use | 3.21 | 3.04 | 8.27 | 3.82 | 3.42 | 2.92 |
| Saw/spoke with clinician in last year | <0.01% | <0.01% | 0.00 | <0.01% | <0.01% | 0.02 |
| Speak English | 50.00 | 0.04 | 0.00 | <0.01% | <0.01% | 0.03 |

^a^ Based on authors’ analysis of NCHS, National Health Interview Surveys, 2013-2018

^b^ Percentages are weighted national estimates of U.S. residents. Age, sex, race, ethnicity, and region had no missing data.
