# Supplementary material for: Disparities in Secure Messaging Uptake Between Patients and Physicians: Longitudinal Analysis of Two National Cross-Sectional Surveys
Source: J Med Internet Res. 2020 May 1;22(5):e12611. doi: 10.2196/12611 (PMC7229528; doi:10.2196/12611)
Supplement: Multimedia Appendix 3 [file jmir_v22i5e12611_app3.docx]

Multimedia Appendix 3.
Prevalence (95% Confidence Interval) of Secure Message Use by Physicians Conducting Ambulatory Care Medical Visits ^a, b^

|  | 2013 | 2016 | *P*-value |
| --- | --- | --- | --- |
| Physician specialty |  |  |  |
| Primary care | 46.56 (42.24, 50.89) | 70.19 (61.86, 78.52) | <0.001 |
| Surgical | 29.96 (25.11, 34.81) | 67.05 (59.49, 74.6) | <0.001 |
| Medical | 36.63 (31.64, 41.61) | 58.45 (50.01, 66.89) | <0.001 |
| Certified health IT |  |  |  |
| Yes | 53.12 (49.61, 56.63) | 75.39 (69.53, 81.26) | <0.001 |
| No | 6.50 (3.82, 9.18) | 20.24 (12.61, 27.87) | <0.001 |
| Solo practice |  |  |  |
| Yes | 26.22 (21.98, 30.46) | 48.17 (38.24, 58.1) | <0.001 |
| No | 48.47 (44.81, 52.13) | 75.05 (69.01, 81.09) | <0.001 |
| Practice ownership |  |  |  |
| Physician or physician group | 35.64 (32.47, 38.82) | 61.26 (54.77, 67.74) | <0.001 |
| Insurance co., health plan or HMO; other health corp. | 62.10 (54.90, 69.29) | 95.07 (93.52, 96.61) | <0.001 |
| Medical/academic center; CHC; other hospital | 52.29 (42.22, 62.35) | 93.37 (85.61, 100) | <0.001 |
| Region |  |  |  |
| Northeast | 31.09 (25.24, 36.94) | 52.38 (40.71, 64.04) | <0.01 |
| Midwest | 42.61 (37.72, 47.49) | 76.77 (67.57, 85.96) | <0.001 |
| South | 41.28 (36.87, 45.7) | 66.93 (56.67, 77.2) | <0.001 |
| West | 47.03 (39.25, 54.81) | 69.54 (58.76, 80.32) | <0.01 |

^a^ Based on authors’ analysis of NCHS, National Ambulatory Medical Care Surveys, 2013-2016

^b^ Percentages are weighted national estimates of ambulatory care medical visits.
